# Radiotherapy combined with anti-PD-1 and TKI for primary cardiac angiosarcoma considering the joint assessment of TLSs and PD-L1: a case report

**DOI:** 10.1186/s13019-024-02752-5

**Published:** 2024-04-09

**Authors:** Shuzhe Deng, Xinxin Yang, Lin He, Qian Zhang, Chunbo Zhao, Hongxue Meng

**Affiliations:** 1 Department of Pathology, Harbin Medical University Cancer Hospital, 150 Haping Road, Harbin, 150086 China; 2 Precision Medical Center, Harbin Medical University Cancer Hospital, Harbin, China; 3 Department of stomatology, Heilongjiang provincial hospital, Harbin, China; 4 Department of Abdominal Radiotherapy, Harbin Medical University Cancer Hospital, Harbin, China; 5 Department of Gastrointestinal Radiation Oncology, Harbin Medical University Cancer Hospital, Harbin, China

**Keywords:** Cardiac angiosarcoma, Tertiary lymphoid structures (TLSs), Programmed cell death protein-1 (PD-1), Tyrosine kinase inhibitors (TKI), Radiotherapy, Programmed cell death-ligand 1 (PD-L1)

## Abstract

**Background:**

Primary cardiac angiosarcoma(PCA) has a low incidence rate and poor prognosis. Currently, no unified clinical treatment standards are available.

**Case presentation:**

We report the case of a 48-year-old man presenting chest tightness, breathlessness, and dyspnea. Imaging and postoperative histopathologic studies confirmed PCA and that the tumor had invaded the entire right atrium. The patient developed progressive disease (PD) during postoperative radiotherapy. We used immunotherapy combined with targeted therapy based on the results of molecular profile and evaluation of tertiary lymphoid structures (TLSs) and programmed cell death-ligand 1 (PD-L1). After treatment, the metastatic lymph nodes of the patient were reduced to a certain extent, indicating that combination therapy was effective.

**Conclusion:**

To the best of our knowledge, this is the first report of radiotherapy combined with anti-PD-1 and tyrosine kinase inhibitors(TKI) for PCA. In addition, this is the first report on immunotherapy for PCA based on new evaluation methods, including TLSs, PD-L1, and genomic profile.

**Supplementary Information:**

The online version contains supplementary material available at 10.1186/s13019-024-02752-5.

## Introduction

Primary cardiac angiosarcoma(PCA) is very rare subgroup of soft tissue sarcomas, which has rapid progression, high recurrence and metastasis rates, strong invasiveness, and poor prognosis [1]. Available treatments mainly include surgery, radiotherapy, and chemotherapy. But seldom eradicate this aggressive tumor. The median survival time is 14 months, implying that patients generally do not achieve long-term survival [2]. Recently, immunotherapy has achieved remarkable results even though data on sarcomas, particularly cardiac angiosarcomas, are scarce [3]. Tertiary lymphoid structures (TLSs) are organized aggregates of immune cells in non-immune organs. The presence of TLSs in a variety of tumors, including sarcomas, can predict the prognosis of patients and the efficacy of immunotherapy [4, 5] and is expected to supplement the immunohistochemical evaluation of programmed cell death-ligand 1 (PD-L1), which together guide immunotherapy. Herein, we report the case of a patient with PCA in detail, including the clinical, imaging, and pathological features. In addition, we identified TLSs in the tissue sections. Moreover, a 5-month comprehensive treatment, including immunotherapy, was administered to the patient by considering the results of genetic testing and PD-L1 scoring, providing support for immunotherapy of cardiac angiosarcoma and the role of TLSs in tumors.

## Case report

**Fig. 1:**
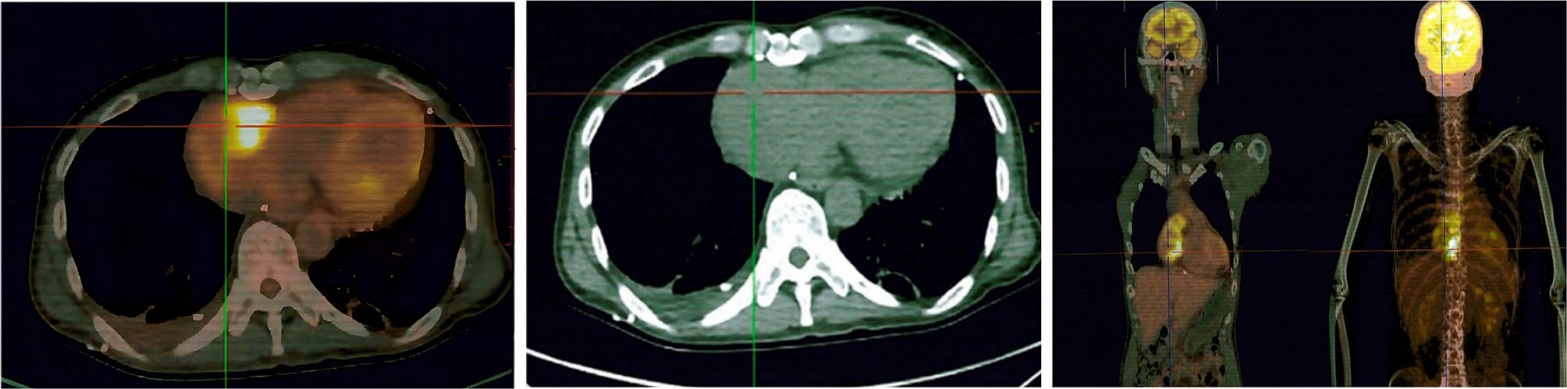
Preoperative 18-fluorodeoxyglucose positron emission tomography-computed tomography(18FDG-PET/CT). A mass was observed in the right atrial area of size approximately 6.5 * 5.9 cm.The maximum standardized uptake value (SUVmax) was 14.2

**Fig. 2:**
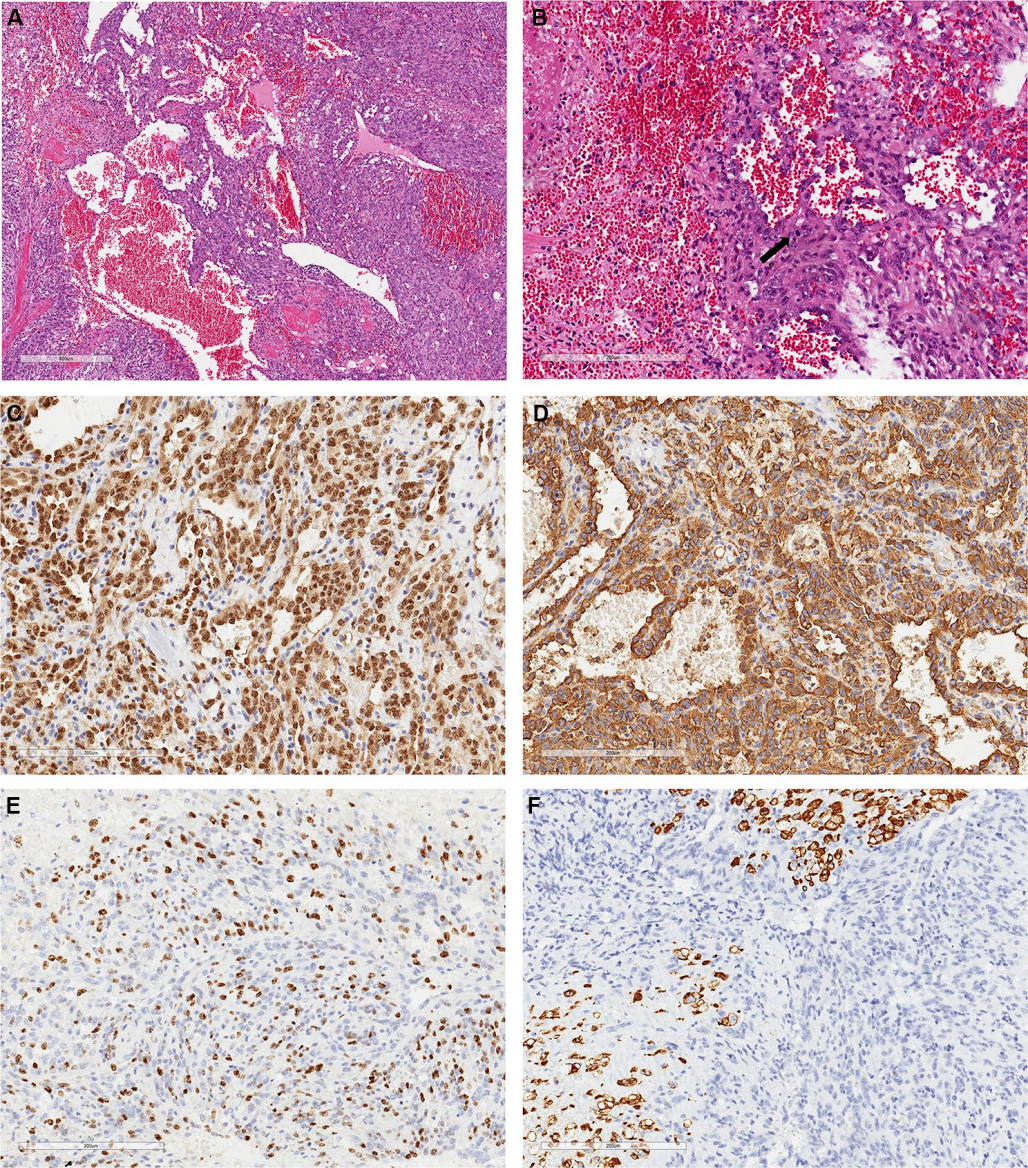
Postoperative pathological diagnosis. (**A**) Hematoxylin and eosin (H&E) staining showed a diffuse growth of atypical spindle and oval cells (× 200). (**B**) Tumor cells interwoven into a network, visible angiogenesis, black arrows shown as a pathological mitotic image (× 400). (**C, D**) Immunohistochemical results revealed tumor cells were ERG(+) and CD31(+) (× 400). (**E**) Ki67 showed that tumor cells had higher proliferative activity (× 400). (**F**) Desmin expression showed that tumor cells destroyed normal myocardial tissue (× 400)

In April 2021, a 48-year-old man was admitted to a local hospital presenting chest tightness and dyspnea without apparent inducement. On April 21, echocardiography showed a 47.2 × 26.1 mm hypoechoic mass in the posterior upper part of the right atrium. On April 23, 18-fluorodeoxyglucose positron emission tomography-computed tomography(18FDG-PET/CT) revealed an area of increased fluorodeoxyglucose(FDG) uptake in the right atrial area (Fig. 1). On April 29, atrial tumor resection and atrial repair were performed under general anesthesia. Unfortunately, the tumor invaded the entire right atrium and reached the level of tricuspid annulus, so it could not be completely removed, and finally only R1 resection was achieved. The postoperative pathological diagnosis was right atrial angiosarcoma (Fig. 2).

**Fig. 3:**
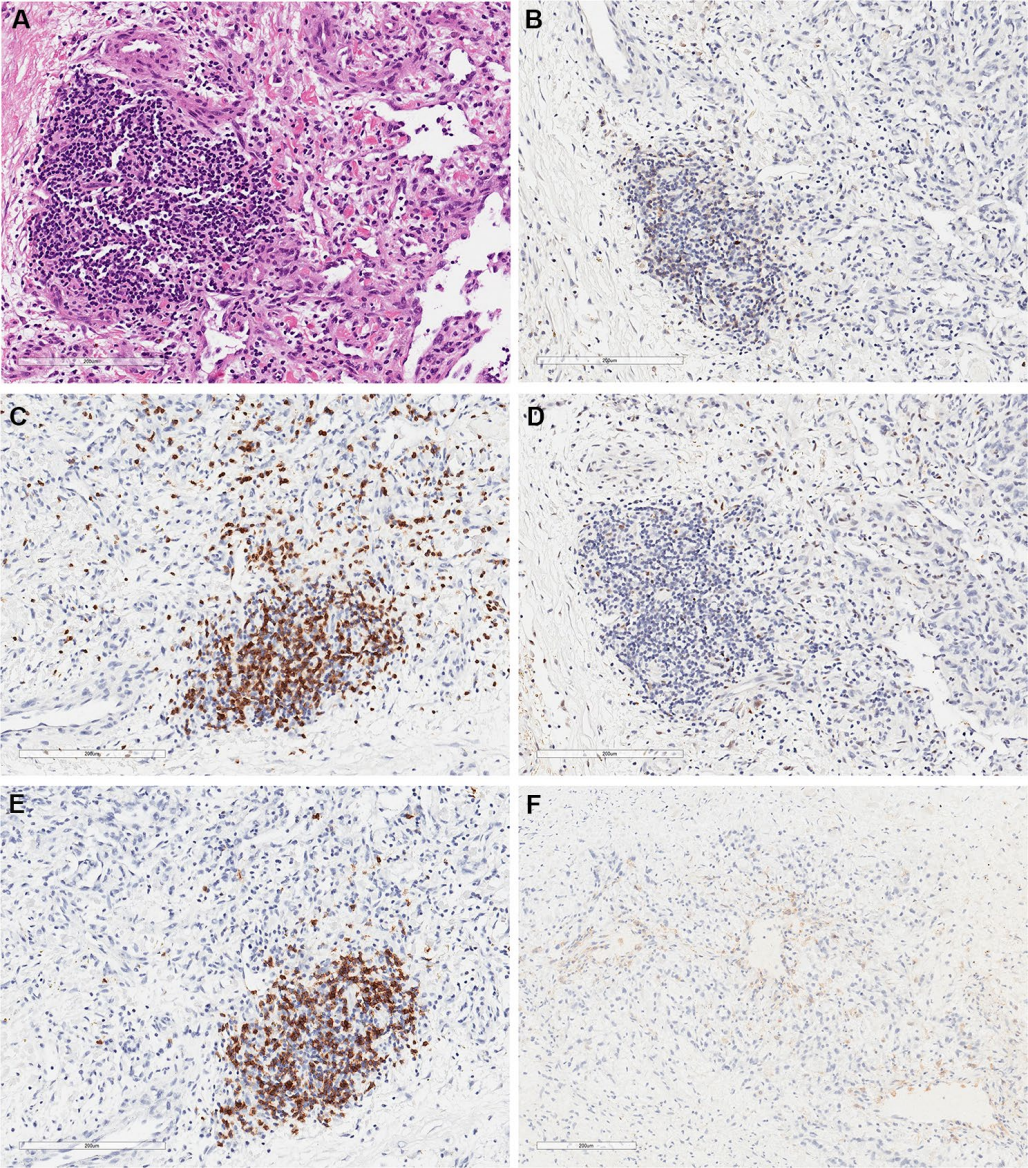
Combined assessment of tertiary lymphoid structures (TLSs) and programmed cell death-ligand 1 (PD-L1). (**A**) Hematoxylin and eosin (H&E) staining of TLSs (× 400). (**B–E**) CD21, CD3, BCL-6, and CD20 were used to show the immature structure and cell composition of the TLSs (× 400). (**F**) Immunohistochemical staining of PD-L1 (× 400)

The next-generation sequencing (NGS) of formalin-fixed and paraffin-embedded (FFPE) tissues revealed mutations in PIK3CA, E545K and TP53, suggesting a poor prognosis. The tumor mutational burden(TMB) was as high as 44.83 mutations/Mb, suggesting that the patient could be a responder to immunotherapy. We evaluated the TLSs on hematoxylin and eosin (H&E) sections and found three recognizable TLSs at the edge of the tumor bed (Fig. 3A). Then we used immunohistochemistry(IHC) to confirm that TLSs are immature (Fig. 3B–E) and PD-L1 combined positive score(CPS) > 1 (Fig. 3F).

**Fig. 4:**
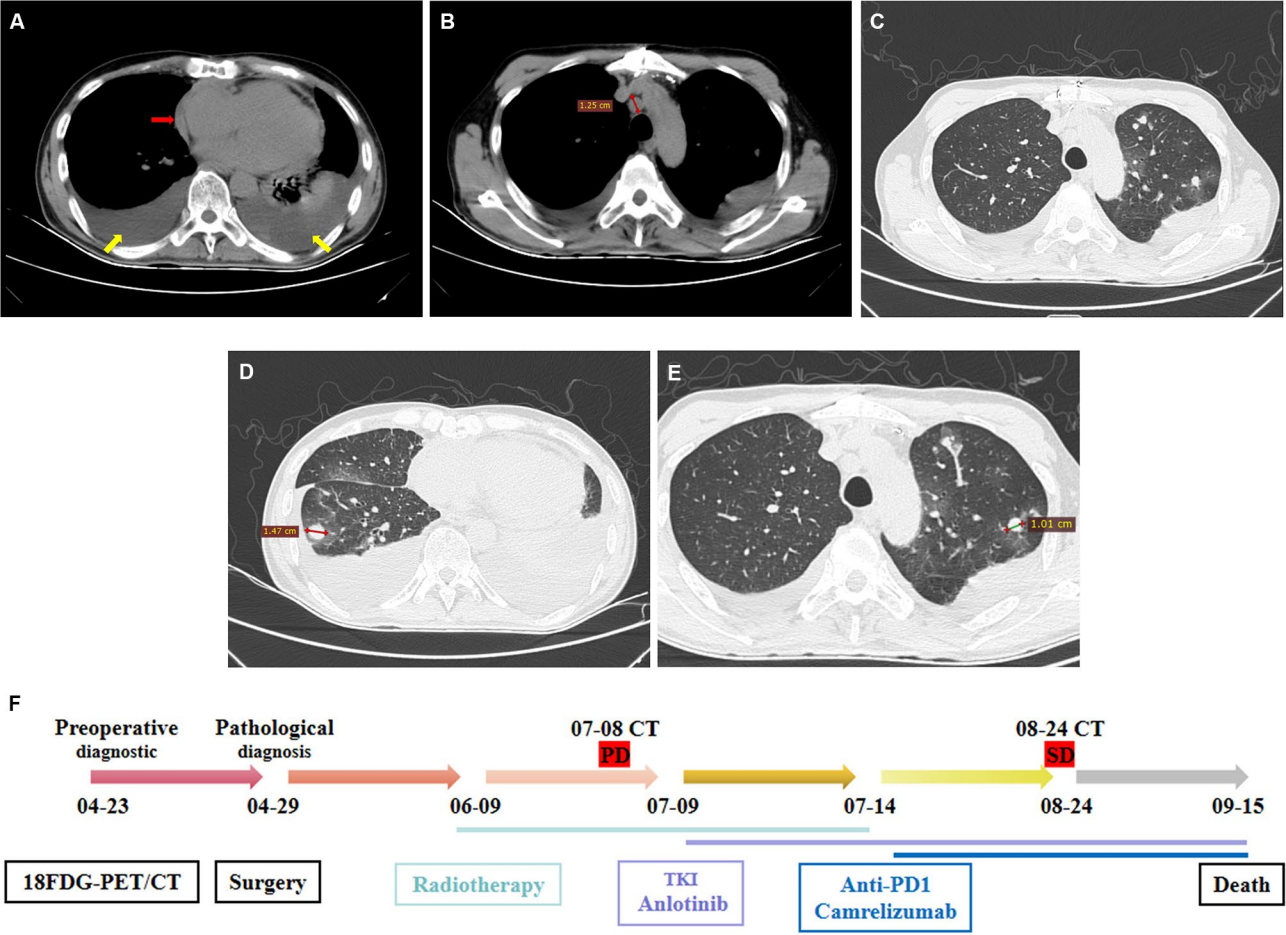
Chest computed tomography (CT) scan to evaluate the efficacy. (**A–D**) Images of disease progression during postoperative radiotherapy. (**A**) Bilateral pleural and pericardial effusion (shown in yellow and red arrows, respectively). (**B**) Station 4R was enlarged with a long diameter of about 1.25 cm. (**C**) Multiple nodules in both lungs (highly suspected metastasis). (**D**) The longest diameter of pulmonary nodules was about 1.47 cm. (**E**) After immunotherapy, bilateral pulmonary nodules could be observed. The longest diameter was approximately 1.01 cm. (**F**) Timeline

From June 9, 2021, to July 14, 2021, postoperative radiotherapy was performed in our hospital. Intensity-modulated radiotherapy conventional segmentation, tumor bed area CTV1 (50 Gy in 25 fractions), and subclinical area CTV2 (40 Gy in 25 fractions). On July 8, a chest computed tomography(CT) of the patient during treatment revealed enlarged mediastinal lymph node and multiple nodules in both lung. The latter was considered to be metastasis, and the maximum diameter was about 1.47 cm (Fig. 4A–D). These findings indicated disease progression (PD) during treatment. On July 9, 10 mg of anlotinib daily oral targeted therapy was administered at our hospital, and 200 mg of camrelizumab immunotherapy on July 14. After discharge, Qizhen capsule was taken orally.The patient was admitted to a local hospital from July 27, to August 5. After admission, 200 mg of camrelizumab was administered intravenously. Anlotinib capsule (10 mg once daily) was prescribed for long-term medication continuously for two weeks, stopped a week, and continued for 21 days for a cycle. On August 24, echocardiography revealed mild pulmonary regurgitation, pericardial effusion, and tachycardia. Enhanced chest CT revealed multiple nodules in both lungs, with the largest diameter of about 1.01 cm (Fig. 4E), passive atelectasis in the left lung because of pleural effusion, and increased pericardial effusion. On September 15, the patient died. The diagnostic process of the patient and staged efficacy evaluation were summarized in Fig. 4F.

## Discussion

The clinical symptoms of primary angiosarcoma are atypical and primarily manifest as arrhythmia, coughing, and dyspnea depend on which side and which cardiac cavity is affected. Because the early stages of the disease are easily ignored, primary angiosarcoma is relatively serious and can progress rapidly [2]. Currently, no standardized treatment for cardiac angiosarcoma is found. Surgery remains the primary treatment for localized PCA [6]. Chemotherapy remains an essential palliative treatment for patients with advanced angiosarcoma who cannot undergo surgery or have distant metastases [7].In addition, neoadjuvant chemotherapy can increase the probability of RO resection and prolong the survival of patients [8]. Unfortunately, our patient underwent surgery soon after being diagnosed by 18FDG-PET/CT without pre-operative and post-operative chemotherapy at a local hospital. For cardiac angiosarcoma, some researchers have applied concurrent adjuvant radiotherapy (50 Gy/2 Gy/25 fractions). No observable adverse reactions during radiotherapy occurred, and the tumor bed remained stable after [9].In our case, postoperative radiotherapy was administered; however, the disease rapidly progressed, and multiple metastases occurred in both lungs. Furthermore, targeted anti-angiogenic drugs and immune checkpoint inhibitors(ICIs) have been applied in clinical practice and achieved good results [10–12].Especially in angiosarcoma, data on the efficacy of immunotherapy are few but consistent in demonstrating excellent antitumour activity. A retrospective study found that patients with visceral angiosarcoma, including cardiac angiosarcoma, treated with pembrolizumab as monotherapy can also obtain progression-free survival (PFS) similar to other systemic treatments [13].

Currently, the value of PD-L1 as a predictive indicator remains unclear. TLSs are structured immune aggregates present in tumor microenvironment(TME), which indicate good clinical outcomes in most cases and can predict immunotherapy efficacy [14]. Therefore, combined with the evaluation of PD-L1 and TLSs, as well as high TMB, we administered tyrosine kinase inhibitors(TKI) combined with programmed cell death protein-1(PD-1) inhibitor carrelizumab to this patient. After treatment, there was a transient stabilization of the disease, and the metastatic lymph nodes were reduced to a certain extent, indicating that treatment had some effect, which was consistent with previous research results [15, 16].

## Conclusion

Unfortunately, the overall survival (OS) of the patient was short. A possible reason for this is palliative surgery [17]. In our case, as mentioned above, preoperative neoadjuvant chemotherapy or complete tumor resection combined with cardiac autotransplantation may give patient the opportunity to receive RO resection, thereby prolonging survival time [18].Furthermore, the disease progressed rapidly, and lung metastasis occured during postoperative radiotherapy. Finally, the results of NGS showed that the mutations of PIK3CA, E545K and TP53 ,suggesting poor prognosis.

Although patient’s survival did not meet our expectations, we applied a new combined assessment of TLSs and PD-L1 in PCA. Based on the results of the above evaluation and genetic testing, we first used combined therapy, including immunotherapy, and achieved a certain effect. In summary, reasonable induction of TLS formation, application of appropriate ICIs, and consideration of dual-target drugs are new ideas for treating PCA in the future and are expected to improve the survival time of patients.

### Electronic supplementary material

Below is the link to the electronic supplementary material.

Supplementary Material 1

Supplementary Material 2

## Data Availability

No datasets were generated or analysed during the current study.

## Abbreviations

PD: Progressive disease
TLSs: Tertiary lymphoid structures
PD-1: Programmed cell death protein-1
PD-L1: Programmed cell death-ligand 1
TKI: Tyrosine kinase inhibitors
TME: Tumor microenvironment
18FDG-PET/CT: 18-fluorodeoxyglucose positron emission tomography-computed tomography
FDG: Fluorodeoxyglucose
CT: Computed tomography
FFPE: Formalin-fixed and paraffin-embedded
H&E: Hematoxylin and eosin
IHC: Immunohistochemistry
NGS: Next-generation sequencing
OS: Overall survival
CPS: Combined positive score
PCA: Primary cardiac angiosarcoma
PFS: Progression-Free Survival
ICIs: Immune checkpoint inhibitors
TMB: Tumor mutational burden
