# Supplementary material for: Radiotherapy combined with anti-PD-1 and TKI for primary cardiac angiosarcoma considering the joint assessment of TLSs and PD-L1: a case report
Source: J Cardiothorac Surg. 2024 Apr 9;19:194. doi: 10.1186/s13019-024-02752-5 (PMC11003096; doi:10.1186/s13019-024-02752-5)
Supplement: Supplementary file 2 — Supplementary Material 2 [file 13019_2024_2752_MOESM2_ESM.doc]

**Radiotherapy combined with anti-PD-1 and TKI for primary cardiac angiosarcoma considering the joint assessment of TLSs and PD-L1：A case report**

List of authors: Shuzhe Deng, Xinxin Yang, Lin He, Chunbo Zhao, Qian Zhang,Hongxue Meng

Full affiliations of all authors and contact details of the corresponding author

**Shuzhe Deng** ,MD：3233@hrbmu.edu.cn

Department of Pathology, Harbin Medical University Cancer Hospital, China.

**Xinxin Yang** ,MD：900212@ hrbmu.edu.cn

Precision Medical Center, Harbin Medical University Cancer Hospital, China.

**Lin He** ,MD：280220862@qq.com

Department of stomatology, Heilongjiang provincial hospital, China.

**Chunbo Zhao** ,MD：zhaochunboTTT@126.com

Department of Gastrointestinal Radiation Oncology, Harbin Medical University Cancer Hospital, China.

**Qian Zhang**, MD: 3236@hrbmu.edu.cn

Department of Abdominal Radiotherapy, Harbin Medical University Cancer Hospital, China.

**Hongxue Meng(corresponding author)** ,MD/Phd：menghongxue@hrbmu.edu.cn

Department of Pathology, Harbin Medical University Cancer Hospital, 150 Haping Road, Harbin 150086, China.

**Abstract**

**Background** Primary cardiac angiosarcoma has a low incidence rate and poor prognosis. Currently, no unified clinical treatment standards are available.

**Case presentation** We report the case of a healthy 48-year-old man presenting chest tightness, breathlessness, and dyspnea. Imaging and postoperative histopathologic studies confirmed primary cardiac angiosarcoma and that the tumor had invaded the entire right atrium. The patient developed progressive disease (PD) during postoperative radiotherapy. We used immunotherapy combined with targeted therapy based on the results of gene detection and evaluation of tertiary lymphoid structures (TLSs) and programmed cell death-ligand 1 (PD-L1). After treatment, the metastatic lymph nodes of the patients were reduced to a certain extent, indicating that immunotherapy was effective.

**Conclusion** To the best of our knowledge, this is the first report of radiotherapy combined with anti-PD-1 and anti-tyrosine kinase inhibitor drugs for primary cardiac angiosarcoma. In addition, this is the first report on immunotherapy for primary cardiac angiosarcoma based on new evaluation methods, including TLSs, PD-L1, and gene detection.

**Keywords** cardiac angiosarcoma, tertiary lymphoid structures (TLSs), programmed cell death protein 1 (PD1), tyrosine kinase inhibitors (TKI), radiotherapy, programmed cell death-ligand 1 (PD-L1)

**Introduction**

Primary cardiac angiosarcoma is rare, which has rapid progression, high recurrence and metastasis rates, strong invasiveness, and poor prognosis[1]. Clinical treatments mainly include surgery, radiotherapy, and chemotherapy. Nevertheless, their curative effect is not ideal. The median survival time is 14 months, implying that patients generally do not achieve long-term survival[2]. Recently, immunotherapy has achieved remarkable results even though data on sarcomas, particularly cardiac angiosarcomas, are scarce[3]. Tertiary lymphoid structures (TLSs) are organized aggregates of immune cells in non-immune organs. The presence of TLSs in a variety of tumors, including sarcomas, can predict the prognosis of patients and the efficacy of immunotherapy [4, 5] and is expected to supplement the evaluation results of programmed cell death-ligand 1 (PD-L1) immunohistochemistry, which together guide immunotherapy. Herein, we report the case of a patient with primary cardiac angiosarcoma in detail, including the clinical, imaging, and pathological features. In addition, we identified TLSs in the tissue sections. Moreover, a 5-month comprehensive treatment, including immunotherapy, was administered to the patients by considering the results of genetic testing and PD-L1 scoring, providing support for immunotherapy of cardiac angiosarcoma and the role of TLSs in tumors.

**Case report**

In April 2021, a 48-year-old man was admitted to a local hospital presenting chest tightness and dyspnea without apparent inducement. On April 21, 2021, echocardiography showed a 47.2 × 26.1 mm hypoechoic mass in the posterior upper part of the right atrium. On April 23, 2021, positron emission tomography-computed tomography (PET-CT) revealed a placeholder in the right atrial area (Fig.1) and inflammation in both lungs (Fig.4e). On April 29, 2021, surgical treatment was performed. The postoperative pathological diagnosis was right atrial angiosarcoma (Fig.2).

Genetic testing of formalin-fixed and paraffin-embedded (FFPE) tissues revealed mutations in PIK3CA E545K and TP53, suggesting a poor prognosis. The tumor mutation load was as high as 44.83 mutations/Mb, suggesting that the patient was the dominant beneficiary of immunotherapy. We evaluated the TLSs on hematoxylin and eosin (H&E) sections and found three recognizable TLSs at the edge of the tumor bed (Fig.3a). Then we used IHC to confirm that TLSs are immature (Fig.3c-f) and PD-L1 combined positive score > 1 (Fig.3b).

From June 9, 2021, to July 14, 2021, postoperative high-risk subclinical radiotherapy was performed in our hospital. Intensity-modulated radiotherapy conventional segmentation, tumor bed area CTV1 (50 Gy in 25 fractions), and subclinical area CTV2 (40 Gy in 25 fractions). On July 8, 2021, a chest CT of the patient during treatment revealed enlarged mediastinal lymph nodes and multiple nodules in both lungs, which were considered metastases (Fig.4a-d). These findings indicated disease progression (PD) during treatment. On July 9, 2021, 10 mg of anrotinib daily oral targeted therapy was administered at our hospital, and 200 mg of camrelizumab immunotherapy on July 14, 2021. After discharge, oral Qizhen capsule righting was initiated.The patient was admitted to a local hospital from July 27, 2021, to August 5, 2021. After admission, 200 mg of camrelizumab was administered intravenously. Anrotinib capsule (10 mg once daily) was prescribed for long-term medication continuously for two weeks, stopped a week, and continued for 21 days for a cycle. On August 24, 2021, Electrocardiographym (ECG) revealed mild pulmonary regurgitation, pericardial effusion, and tachycardia. Enhanced chest CT revealed multiple nodules in both lungs with a larger diameter of approximately 1.0 cm (Fig.4f), partial atelectasis in the left lung, bilateral pleural effusion with an incomplete expansion of both lungs, and increased pericardial effusion. On September 15, 2021, the patient died. The diagnostic process of the patient and staged efficacy evaluation is summarized in Fig.4g.

**Discussion**

The clinical symptoms of primary angiosarcoma are atypical and primarily manifest as arrhythmia, coughing, and dyspnea. Because the early stages of the disease are easily ignored, primary angiosarcoma is highly malignant and can progress rapidly[2]. Currently, no standardized treatment for cardiac angiosarcoma is found. Surgery remains the primary treatment for localized primary cardiac angiosarcomas[6]. Chemotherapy remains an essential palliative treatment for patients with advanced angiosarcoma who cannot undergo surgery or have distant metastases[7]. For cardiac angiosarcoma, some researchers have applied concurrent adjuvant radiotherapy (50 Gy/2 Gy/25 min). No observable adverse reactions during radiotherapy occurred, and the local lesions remained stable after[8]. However, some studies have reported that postoperative radiotherapy is not beneficial[9]. In our case, postoperative radiotherapy was administered; however, the disease was not effectively controlled, and multiple metastases occurred in both lungs. In addition, targeted anti-vascular drugs and immune checkpoint inhibitors have been applied in clinical practice and achieved good results[10-12].

Currently, the value of PD-L1 positivity as a prognostic indicator remains unclear. TLSs are structured immune aggregates present in the TME that are mainly composed of lymphocytes. Mature TLSs indicate good clinical outcomes in most cases and can predict immunotherapy efficacy[13]. Therefore, combined with the evaluation of PD-L1 and TLSs and the results of high tumor mutation load, we administered the PD-1 inhibitor carrelizumab to this patient. After treatment, the disease progression was stable, and the metastatic lymph nodes were reduced to a certain extent, indicating that immunotherapy had some effect.

**Conclusion**

Unfortunately, the overall survival (OS) of the patient was short. A possible reason for this is palliative surgery. Furthermore, radiotherapy also had certain damage to the myocardium. Although TLSs existed in H&E sections of the patients, they were immature and located in the paracancerous areas. However, the PD-L1 staining score had limitations. Therefore, the effectiveness of monoclonal antibody immunotherapy drugs administered to the patients may be insufficient. Finally, resistance to immune-monotherapy was considered[14]. Although patient survival did not meet our expectations, we applied a new combined assessment of TLSs and PD-L1 in cardiac angiosarcoma. Based on the results of genetic testing, we first used combined therapy, including immunotherapy, and achieved a certain effect, filling the gap in TLSs and combined immunotherapy in cardiac angiosarcoma. In summary, reasonable induction of mature TLS formation, application of appropriate immune checkpoint inhibitors, and consideration of dual-target drugs are new ideas for treating

primary cardiac angiosarcoma in the future and are expected to improve the survival time of patients.

**Abbreviations**

| PD | Progressive disease |
| --- | --- |
| TLSs | Tertiary lymphoid structures |
| PD1 | Programmed cell death protein 1 |
| PD-L1 | Programmed cell death-ligand 1 |
| TKI | Tyrosine kinase inhibitors |
| TME | Tumor microenvironment |
| PET-CT | Positron emission tomography-computed tomography |
| CT | Chest computed tomography |
| FFPE | Formalin-fixed and paraffin-embedded |
| H&E | Hematoxylin and eosin |
| IHC | Immunohistochemistry |
| ECG | Electrocardiographym |
| OS | Overall survival |

**Authors’ contributions**

Shuzhe Deng reviewed the literature and wrote the first draft. Xinxin Yang, Lin He, Chunbo Zhao，Qian Zhang revised and interpreted the information related. Hongxue Meng administrated and finalized the manuscript. All authors read and approved the final manuscript.

**Funding**

This work was supported by grants from the National Nature Science Foundation of China (82072985), Postdoctoral Scientific Research Developmental Fund of Heilongjiang Province (LBH-Q18076), the N10 Found project of Harbin Medical University Cancer Hospital (2017-03), Wu-Jieping Medical Foundation (320.6750.19089-22,320.6750.19089-48), Beijing Medical Award Foundation (YXJL-2019-1416-0069), Key R&D Program of Heilongjiang Province (GY2023JD0002), Hai Yan Youth Fund of Harbin Medical University Cancer Hospital (JJQN2021-02), the Fundamental Research Funds for the Provincial Universities(2021-KYYWF-0253), Natural Science Foundation of Heilongjiang Province（LH2022H065)，Natural Science Foundation of Heilongjiang Province (LH2021H066)，Scientific research project of the HeiLongjiang Provincial Health Commission(20210808020126).

**Data Availability**

Not applicable.

**Declarations**

**Ethics approval and consent to participate**

Not applicable.

**Consent for publication**

Patient consented for participation in research.

**Competing interests**

The authors declare no competing interests.

**References**

1. Tyebally S, Chen D, Bhattacharyya S, et al. Cardiac Tumors: JACC CardioOncology State-of-the-Art Review. JACC CardioOncol. 2020;2(2):293-311.

2. Patel SD, Peterson A, Bartczak A, et al. Primary cardiac angiosarcoma - a review. Med Sci Monit. 2014;20:103-9.

3. Gavrielatou N, Doumas S, Economopoulou P, et al. Biomarkers for immunotherapy response in head and neck cancer. Cancer Treat Rev. 2020;84:101977.

4. Petitprez F, de Reynies A, Keung EZ, et al. B cells are associated with survival and immunotherapy response in sarcoma. Nature. 2020;577(7791):556-60.

5. Liang H, Zhang Z, Guan Z, et al. Follicle-like tertiary lymphoid structures: A potential biomarker for prognosis and immunotherapy response in patients with laryngeal squamous cell carcinoma. Frontiers in Immunology. 2023;14.

6. Young RJ, Brown NJ, Reed MW, et al. Angiosarcoma. Lancet Oncol. 2010;11(10):983-91.

7. Tap WD, Jones RL, Van Tine BA, et al. Olaratumab and doxorubicin versus doxorubicin alone for treatment of soft-tissue sarcoma: an open-label phase 1b and randomised phase 2 trial. Lancet. 2016;388(10043):488-97.

8. Fang X, Zheng S. Primary cardiac angiosarcoma: a case report. J Int Med Res. 2021;49(8):3000605211033261.

9. Abraham JA, Hornicek FJ, Kaufman AM, et al. Treatment and outcome of 82 patients with angiosarcoma. Ann Surg Oncol. 2007;14(6):1953-67.

10. Chi Y, Fang Z, Hong X, et al. Safety and Efficacy of Anlotinib, a Multikinase Angiogenesis Inhibitor, in Patients with Refractory Metastatic Soft-Tissue Sarcoma. Clin Cancer Res. 2018;24(21):5233-8.

11. Somaiah N, Conley AP, Parra ER, et al. Durvalumab plus tremelimumab in advanced or metastatic soft tissue and bone sarcomas: a single-centre phase 2 trial. Lancet Oncol. 2022;23(9):1156-66.

12. D'Angelo SP, Richards AL, Conley AP, et al. Pilot study of bempegaldesleukin in combination with nivolumab in patients with metastatic sarcoma. Nat Commun. 2022;13(1):3477.

13. Sautes-Fridman C, Petitprez F, Calderaro J, et al. Tertiary lymphoid structures in the era of cancer immunotherapy. Nat Rev Cancer. 2019;19(6):307-25.

14. Vesely MD, Zhang T, Chen L. Resistance Mechanisms to Anti-PD Cancer Immunotherapy. Annu Rev Immunol. 2022;40:45-74.

**Figure legends**

Figure 1．Preoperative PET-CT diagnosis.

A mass shadow was observed in the right atrial area of size approximately 6.5 * 5.9 cm. The right atrium was not well displayed, and the local metabolism of the lesion increased. The maximum standardized uptake value (SUVmax) was 14.2. A slightly increased lymph node was seen in the mediastinum, with an SUVmax of 7.2.

Figure 2．Postoperative pathological diagnosis.

(a). Hematoxylin and eosin (H&E) staining showed a diffuse growth of atypical spindle and oval cells (× 200).

(b). Tumor cells interwoven into a network, visible angiogenesis, black arrows shown as a pathological mitotic image (× 400).

(c-d). Immunohistochemical results revealed tumor cells were ERG(+) and CD31(+) (× 400).

(e). Ki67 showed that tumor cells had higher proliferative activity (× 400).

(f). Desmin expression showed that tumor cells destroyed normal myocardial tissue (× 400).

Figure 3．Combined assessment of tertiary lymphoid structures (TLSs) and programmed cell death-ligand 1 (PD-L1).

(a). Hematoxylin and eosin (H&E) staining of TLSs (× 400).

(b). Immunohistochemical staining of PD-L1 (× 400).

(c-f). CD21, CD3, BCL-6, and CD20 were used to show the immature structure and cell composition of the TLSs (× 400).

Figure 4 .Chest computed tomography (CT) scan to evaluate the efficacy.

(a-d). Images of disease progression during postoperative radiotherapy.

(a). Bilateral pleural and pericardial effusion (shown in yellow and red arrows, respectively).

(b). Mediastinal lymph node was enlarged with a long diameter of about 1.25 cm.

(c). Multiple nodules in both lungs (highly suspected metastasis).

(d). The longest diameter of pulmonary nodules was about 1.47 cm.

(e). Preoperative positron emission tomography-computed tomography (PET-CT) showed multiple patchy cords and consolidation in both lungs, showing inflammatory changes.

(f). After immunotherapy, bilateral pulmonary nodules could be observed. The longest diameter was approximately 1.01 cm.

(g). The complete process of diagnosis and treatment.
